# Effectiveness of LODS, OASIS, and SAPS II to predict in-hospital mortality for intensive care patients with ST elevation myocardial infarction

**DOI:** 10.1038/s41598-021-03397-3

**Published:** 2021-12-13

**Authors:** Liang Wang, Zhengwei Zhang, Tianyang Hu

**Affiliations:** 1 Department of Cardiology, The Second Affiliated Hospital, Chongqing Medical University, 74 Linjiang Road, Yuzhong District, Chongqing, 400010 China; 2 Department of Critical Care Medicine, Chengdu Second People’s Hospital, Chengdu, China

**Keywords:** Medical research, Biomarkers, Outcomes research

## Abstract

The relationship between three scoring systems (LODS, OASIS, and SAPS II) and in-hospital mortality of intensive care patients with ST segment elevation myocardial infarction (STEMI) is currently inconclusive. The baseline data, LODS score, OASIS score, SAPS II score, and in-hospital prognosis of intensive care patients with STEMI were retrieved from the Medical Information Mart for Intensive Care IV database. Propensity score matching analysis was performed to reduce bias. Receiver operating characteristic curves (ROC) were drawn for the three scoring systems, and comparisons between the areas under the ROC curves (AUC) were conducted. Decision curve analysis (DCA) was performed to determine the net benefits of the three scoring systems. LODS and SAPS II were independent risk factors for in-hospital mortality. For the study cohort, the AUCs of LODS, OASIS, SAPS II were 0.867, 0.827, and 0.894; after PSM, the AUCs of LODS, OASIS, SAPS II were 0.877, 0.821, and 0.881. A stratified analysis of the patients who underwent percutaneous coronary intervention/coronary artery bypass grafting (PCI/CABG) or not was conducted. In the PCI/CABG group, the AUCs of LODS, OASIS, SAPS II were 0.853, 0.825, and 0.867, while in the non-PCI/CABG group, the AUCs of LODS, OASIS, SAPS II were 0.857, 0.804, and 0.897. The results of the Z test suggest that the predictive value of LODS and SAPS II was not statistically different, but both were higher than OASIS. According to the DCA, the net clinical benefit of LODS was the greatest. LODS and SAPS II have excellent predictive value, and in most cases, both were higher than OASIS. With a more concise composition and greater clinical benefit, LODS may be a better predictor of in-hospital mortality for intensive care patients with STEMI.

## Introduction

Acute myocardial infarction (AMI) is one of the leading causes of global cardiovascular burden, among which ST elevation myocardial infarction (STEMI) has attracted much attention of clinicians due to its high mortality and morbidity^1,2^. In the past decade, the widespread use of percutaneous coronary intervention (PCI) has significantly increased the survival chances of STEMI patients^3^, however, the survival rate is still not within the ideal range. According to reports from recent studies^4,5^, the residual in-hospital mortality of STEMI ranges from 5–10%. Risk classification is critical for STEMI and can be used for selecting treatment regimens as well as planning hospital discharge, especially for patients admitted to the intensive care unit (ICU). But currently, there is no recognized tool for predicting in-hospital mortality for intensive care patients with STEMI. Thus, it is particularly important to find an effective tool.

Several scoring systems have been proven effective for predicting mortality in intensive care patients. The Logistic Organ Dysfunction System (LODS) is an organ dysfunction scoring system and permits the calculation of predicted mortality based on the organ dysfunction score on the day of ICU admission^6^. The LODS score has been effectively used to predict the mortality of intensive care patients with sepsis and patients in neurological ICU^7,8^. The Oxford Acute Severity of Illness Score (OASIS) was proposed in 2013 by machine-learning algorithms, and predictive models of ICU mortality using OASIS achieved an area under the receiver operating characteristic curve of 0.88 and calibrated well^9^. The Simplified Acute Physiology Score (SAPS II) provides an estimate of the risk of mortality without having to specify a primary diagnosis and was regarded as a starting point for evaluation of the efficiency of ICU^10^. In this study, we aimed to explore the performance of the above three scoring systems in predicting the in-hospital mortality of intensive care patients with STEMI, to provide valuable clues to clinical practice.

## Methods

### Database

This retrospective study was designed based on the critical care database, Medical Information Mart for Intensive Care IV (MIMIC-IV, https://mimic.mit.edu/). The database contains real hospital stays between 2008 and 2019 for patients admitted to a tertiary academic medical center in Boston, MA, USA. According to the official requirements of the database, investigators must complete the "Protect Human Research Participants" exam on the National Institutes of Health website and sign a data use agreement before granting access^11^. One author (Tianyang Hu) passed the exam (record ID: 37474354) and obtained access to the database.

This study was conducted in accordance with the Declaration of Helsinki. The establishment of the MIMIC-IV database was approved by the Massachusetts Institute of Technology and Beth Israel Deaconess Medical Center, and consent was obtained for the original data collection. The patients in the database are anonymous, therefore, the ethical approval statement and the need for informed consent were waived for this study.

### Study population and data extraction

All intensive care patients diagnosed with STEMI were screened and identified by the “long_title” in the “d_icd_diagnoses” table of MIMIC-IV database. Since one patient may be admitted to the ICU multiple times, we only included the first ICU stay for each patient. Navicat Premium software (v15.0) was used to extract the following data of the included patients from MIMIC-IV database (v1.0): age, gender, length of ICU stay, length of hospital stay, coexisting comorbidities (congestive heart failure, peripheral vascular disease, cerebrovascular disease, chronic pulmonary disease, rheumatic disease, peptic ulcer disease, liver disease, diabetes, renal disease, malignant cancer, hypertension, and obesity), laboratory tests (red blood cell, white blood cell, platelets, hemoglobin, anion gap, blood urea nitrogen, creatinine, and INR), vital signs (heart rate, systolic blood pressure, diastolic blood pressure, mean blood pressure, respiratory rate, temperature, and SpO_2_), and scoring systems (LODS, OASIS, and SAPS II). PCI is the standard interventional treatment modality for patients with STEMI^12^, also was proved to be effective on one-year mortality in non-ST-segment elevation myocardial infarction (NSTEMI) patients^13^. Approximately 20–30% of patients are not eligible for PCI and require surgical intervention, that is, coronary artery bypass grafting (CABG)^14,15^. Therefore, we additionally investigated whether the included patients underwent PCI or CABG.

### Statistical analysis

The Kolmogorov–Smirnov test was used to assess the normality of continuous variables. Normally distributed variables were expressed as mean ± standard deviation (M ± SD), and the independent sample t-test was used for comparison; if the distribution was abnormal, continuous variables were expressed as the median with interquartile range (IQR), and Wilcoxon rank-sum test was used for comparison. Categorical variables were expressed as numbers and percentages and compared using the Chi-square test. Binomial logistic regression analysis of the three scoring systems for in-hospital mortality in intensive care patients with STEMI was conducted to adjust the results of the statistical analysis for potential confounding factors by selecting appropriate variables closely related to the condition of STEMI. Variables with *P* values < 0.1 in univariate analysis were included in multivariate analysis. Z test was used to compare the predictive value of the three scoring systems by comparing the area under curves (AUC) of the receiver operating characteristic curves (ROC).

Propensity score matching (PSM) analysis was performed to reduce bias between the in-hospital death group and survival group based on the following variables: age, gender, length of hospital stay, and underwent PCI/CABG or not. The propensity scores were calculated by a logistic regression model, and the PSM analysis was performed using a 1:1 nearest neighbor matching algorithm with a caliper of 0.02 without replacement.

We also performed the decision curve analysis^16–18^ (DCA), a method for evaluating alternative diagnostic and prognostic strategies, to explore the net benefits between the three scoring systems and intensive care patients with STEMI. The concept of the DCA is generally combining accuracy measures and clinical applicability by integrating clinical consequences associated with a test result. The “net benefit” is calculated by the difference between the proportion of relative harms of false positives and false negatives weighted by the odds of the selected threshold for high-risk designation, therefore the difference between the expected benefit and the expected harm. If it is predicted that the outcome event may occur with a certain probability and the emergency intervention measures (for patients with STEMI, they should be revascularization, that is, PCI or CABG) are immediately carried out at the same time, the use of the scoring system corresponding to the largest area under the decision curve will have the greatest clinical benefit.

All analyses were conducted with SPSS software (v26.0; IBM, Armonk, NY), MedCalc statistical software (v19.6.1; MedCalc Software Co., Ltd., Ostend, Belgium), and R software (v4.0.3, CRAN). The Z test was performed using MedCalc statistical software by the method of Delong et al.^19^. DCA was performed by the "rmda" package of R software. *P* value < 0.05 was considered statistically significant.

## Results

### Baseline characteristics

**Figure 1:**
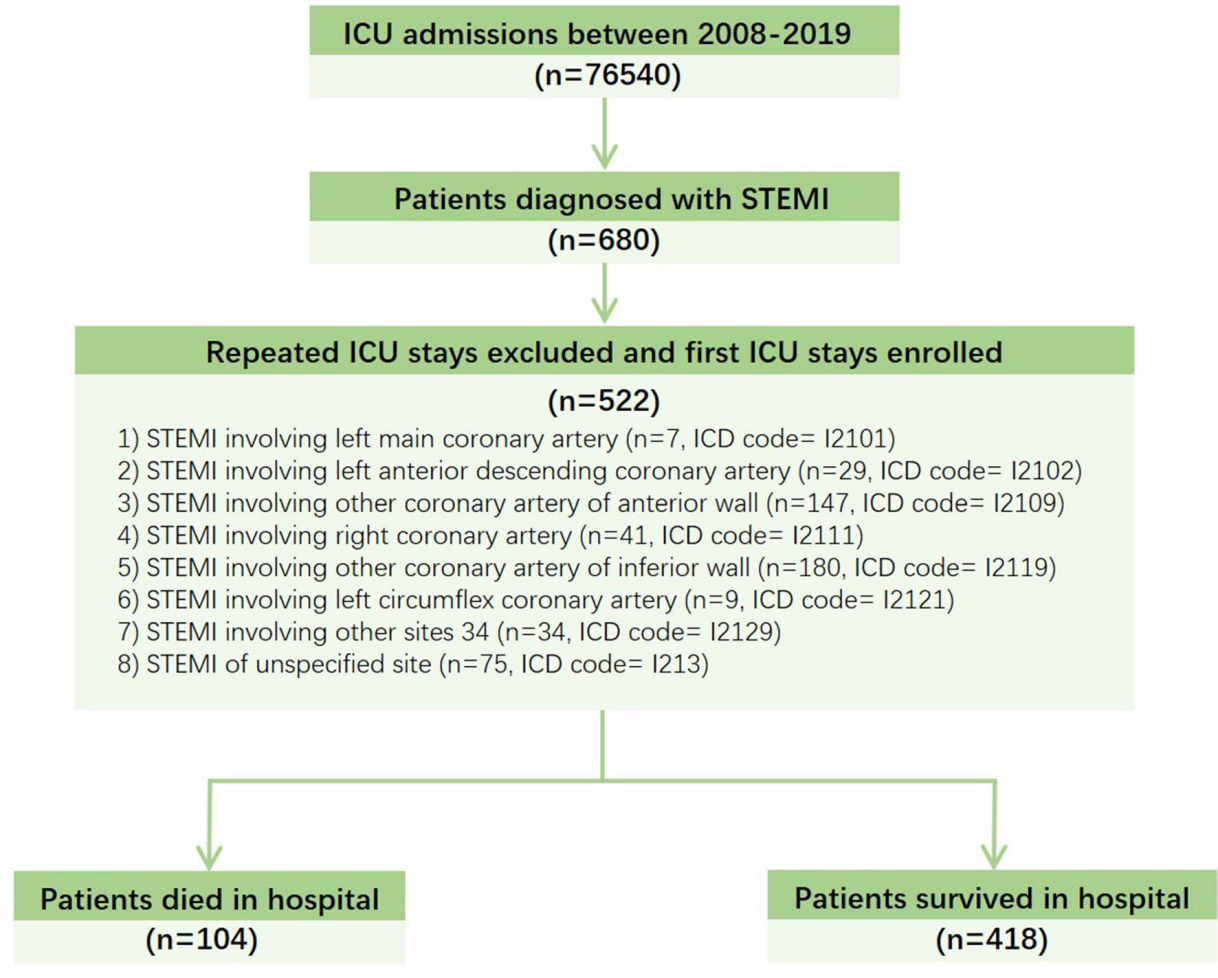
Flowchart of study cohort. *ICU* intensive care unit, *STEMI* ST elevation myocardial infarction, *ICD* International Classification of Disease, 10th Revision.

76,540 ICU admissions were contained in MIMIC-IV database, and finally, 522 patients were enrolled in this study (of which 104 died and 418 survived in hospital, Fig. 1). The length of hospital stay in the survival group death group was longer than in the death group (*P* < 0.001). Age, the level of white blood cell, anion gap, blood urea nitrogen, creatinine, INR, heart rate, and respiratory rate, SAPS II score, LODS score, and OASIS score in the death group was significantly higher than in the survival group (*P* < 0.001 for all), while the level of red blood cell, platelets, hemoglobin, systolic/diastolic/mean blood pressure, and temperature in the survival group was significantly higher than in the death group (*P* < 0.05 for all). There were no significant differences in length of ICU stay and the level of saturation of peripheral oxygen between the two groups. The prevalence of peripheral vascular disease, diabetes, and renal disease was significantly higher in the death group (*P* < 0.05 for all), while the prevalence of hypertension and the proportion of patients who underwent PCI/CABG were significantly higher in the survival group (*P* < 0.05 for all). The baseline characteristics of the study population are presented in Table 1. After propensity score matching, the matching variables are balanced and comparable between the two groups (*P* > 0.05 for all, see *P*_*psm*_ in Table 1 for details), resulting 94 patients in the death group and 94 patients in the survival group.

**Table 1:** Demographic data of the study population.

Characteristics	Death group	Survival group	*P*	*P*_*psm*_
N (sample size)	104	418		
**Baseline variables**				
Age (year)	74.0[67.0–82.5]	68.0[58.0–76.0]	0.000	0.438
Gender (Male)	57(54.8)	284(67.9)	0.012	0.556
LOS ICU (day)	2.0[1.1–5.9]	2.0[1.1–3.2]	0.562	
LOS hospital (day)	3.1[1.3–7.8]	5.1[3.1–9.3]	0.000	0.438
**Coexisting comorbidities**				
Congestive heart failure	59(56.7)	194(46.4)	0.060	
Peripheral vascular disease	18(17.3)	40(9.6)	0.035	
Cerebrovascular disease	13(12.5)	36(8.6)	0.258	
Chronic pulmonary disease	18(17.3)	59(14.1)	0.440	
Rheumatic disease	3(2.9)	11(2.6)	1.000	
Peptic ulcer disease	1(0.9)	6(1.4)	1.000	
Liver disease	5(4.8)	14(3.3)	0.556	
Diabetes	44(42.3)	123(29.4)	0.012	
Renal disease	36(34.6)	69(16.5)	0.000	
Malignant cancer	7(6.7)	17(4.1)	0.292	
Hypertension	24(23.1)	160(38.3)	0.004	
Obesity	12(11.5)	31(7.4)	0.168	
**Laboratory tests**				
Red blood cell(10^12^/L)	3.7 ± 0.7	4.1 ± 0.7	0.000	
White blood cell (10^9^/L)	15.7[11.9–20.0]	11.4[9.0–15.2]	0.000	
Platelets(10^9^/L)	192.3[151.1–230.3]	207.0[167.7–255.5]	0.004	
Hemoglobin(g/dL)	11.0 ± 2.1	12.2 ± 2.0	0.000	
Anion gap(mmol/L)	20.0[16.5–23.0]	15.0[13.0–17.5]	0.000	
BUN (mmol/L)	34.5[27.0–51.5]	17.0[13.5–22.0]	0.000	
Creatinine(ng/dL)	1.9[1.4–2.6]	0.9[0.8–1.2]	0.000	
INR	1.5[1.2–1.9]	1.2[1.1–1.3]	0.000	
**Vital signs**				
Heart rate(bpm)	85.2[72.7–99.0]	77.7[70.1–85.9]	0.000	
SBP (mmHg)	104.7[96.0–113.4]	113.5[105.5–122.9]	0.000	
DBP (mmHg)	61.8 ± 10.1	67.4 ± 10.5	0.000	
MBP (mmHg)	74.6[69.7–82.4]	81.5[75.1–88.4]	0.000	
Respiratory rate(cpm)	21.2[19.0–24.5]	19.0[17.2–21.0]	0.000	
Temperature(℃)	36.7[36.4–37.1]	36.8[36.7–37.0]	0.002	
SpO_2_(%)	96.5[92.5–98.3]	96.7[95.6–97.7]	0.348	
**Scoring systems**				
LODS	11.0[6.5–13.0]	2.0[1.0–5.0]	0.000	
OASIS	45.0[35.0–50.0]	28.0[22.0–35.0]	0.000	
SAPS II	55.0[43.0–69.0]	29.0[23.0–38.0]	0.000	
**PCI/CABG**	41(39.4)	313(74.9)	0.000	0.883

*LOS* Length of Stay, *ICU* Intensive Care Unit, *BUN* Blood Urea Nitrogen, *INR* International Normalized Ratio, *bpm* beat per minute, *SBP* Systolic Blood Pressure, *DBP* Diastolic Blood Pressure, *MBP* Mean Blood Pressure, *cpm* count per minute, *SpO*_*2*_ Saturation of Peripheral Oxygen, *LODS* Logistic Organ Dysfunction System, *OASIS* Oxford Acute Severity of Illness Score, *SAPS II* Simplified Acute Physiology Score, *PCI* Percutaneous Coronary Intervention, *CABG* Coronary Artery Bypass Grafting.

### Logistic regression analysis

The three scoring systems (LODS, OASIS, and SAPS II) were all risk factors for in-hospital mortality in intensive care patients with STEMI (*P* < 0.001 for all) before adjustment. After adjustment, gender, heart rate, and underwent PCI/CABG or not were all independent risk factors for in-hospital mortality; In addition, LODS score and SAPS II were independent risk factors (OR: 1.447, 95% CI 1.208–1.732, *P* = 0.002; OR: 1.060, 95% CI 1.018–1.103, *P* = 0.004) for in-hospital mortality, while OASIS was not correlated with the mortality (OR: 0.962, 95% CI 0.912–1.015, *P* = 0.158) (Table 2).

**Table 2:** Binomial Logistic regression analysis for in-hospital mortality among intensive care patients with STEMI including the three scoring systems.

Variable	Univariable		Multivariable	
	OR (95% CI)	*P*	OR (95% CI)	*P*
Age	1.033(1.015–1.050)	0.000	1.022(0.993–1.052)	0.136
Gender (male)	0.572(0.369–0.886)	0.012	0.524(0.261–1.050)	0.068
LOS hospital (day)	0.946(0.907–0.986)	0.008	0.859(0.802–0.919)	0.000
Congestive heart failure	1.514(0.982–2.334)	0.060	0.954(0.458–1.987)	0.900
Hypertension	0.484(0.294–0.795)	0.004	0.448(0.183–1.098)	0.079
Heart rate(bpm)	1.036(1.021–1.051)	0.000	1.050(1.027–1.074)	0.000
MBP (mmHg)	0.921(0.898–0.945)	0.000	0.973(0.939–1.008)	0.132
LODS	1.523(1.412–1.642)	0.000	1.447(1.208–1.732)	0.000
OASIS	1.146(1.115–1.177)	0.000	0.962(0.912–1.015)	0.158
SAPS II	1.115(1.092–1.138)	0.000	1.060(1.018–1.103)	0.004
PCI/CABG	0.218(0.139–0.343)	0.000	0.453(0.232–0.886)	0.021

*STEMI* ST segment elevation myocardial infarction, *LOS* Length of Stay, *bpm* beat per minute, *MBP* Mean Blood Pressure, *LODS* Logistic Organ Dysfunction System, *OASIS* Oxford Acute Severity of Illness Score, *SAPS II* Simplified Acute Physiology Score, *PCI* Percutaneous Coronary Intervention, *CABG* Coronary Artery Bypass Grafting.

### Comparison of ROC curves

**Figure 2:**
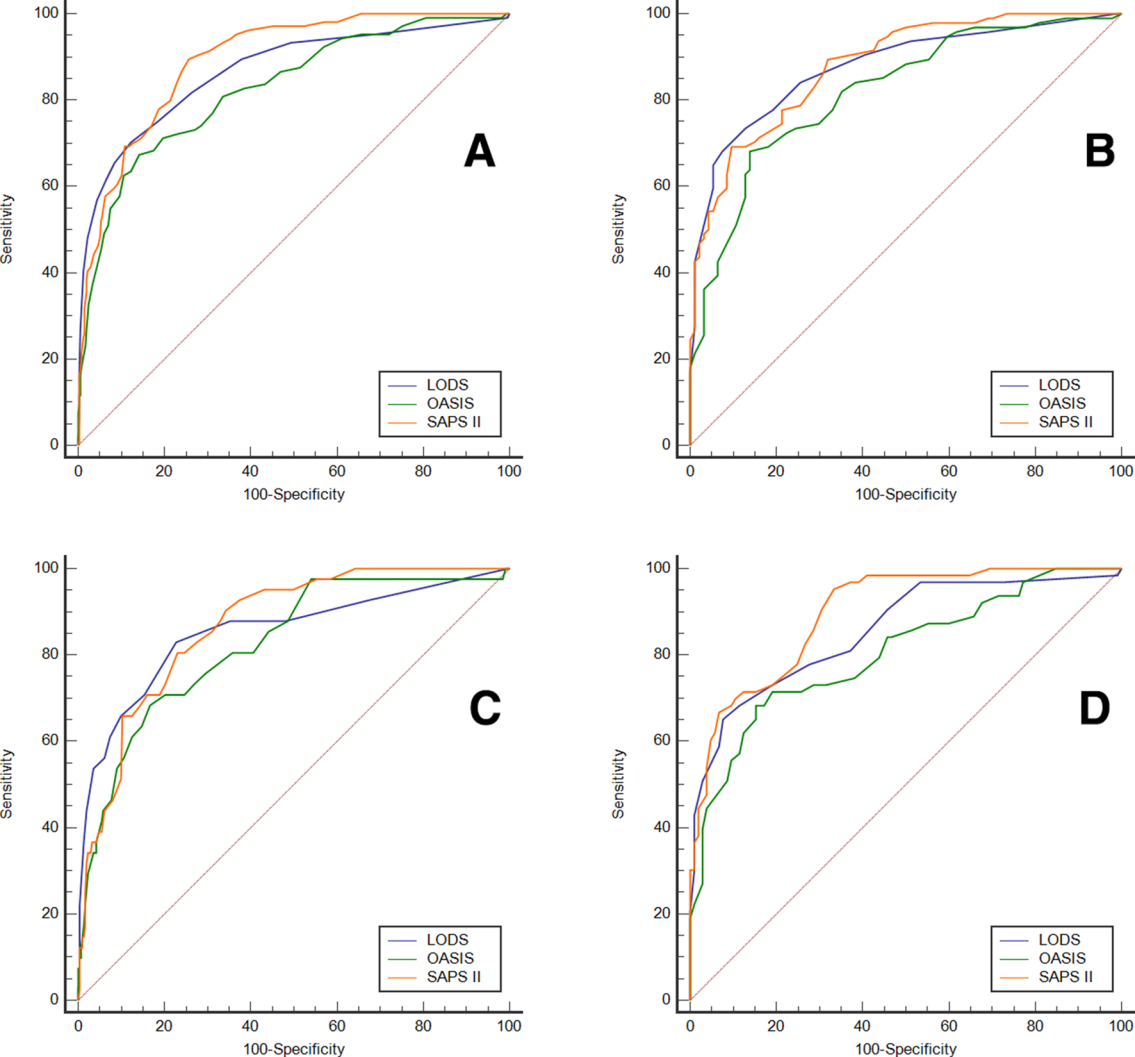
(**A**) ROC curves of the scoring systems for the study cohort (before propensity score matching); (**B**) ROC curves of the scoring systems for the study cohort (after propensity score matching); (**C**) ROC curves of the scoring systems for the patients underwent PCI/CABG; (**D**) ROC curves of the scoring systems for the patients without PCI/CABG.

Before PSM, the AUCs of LODS, OASIS, SAPS II were 0.867, 0.827, and 0.894 for the study cohort, respectively (Fig. 2A). The AUCs were compared, resulting in LODS vs OASIS (Z = 2.365, *P* = 0.018), LODS vs SAPS II (Z = 1.846, *P* = 0.065), and OASIS vs SAPS II (Z = 3.551, P = 0.0004). The cut-off value corresponding to Youden’s index was selected as the optimal cut-off value for predicting in-hospital mortality. SAPS II had the highest sensitivity (89.42%) and Youden’s index (0.6382), while LODS had the highest specificity (87.80%). The results are presented in Table 3. After PSM, the AUCs of LODS, OASIS, SAPS II were 0.877, 0.821, and 0.881, respectively (Fig. 2B). The results of AUCs comparisons were LODS vs OASIS (Z = 2.477, *P* = 0.013), LODS vs SAPS II (Z = 0.210, *P* = 0.833), and OASIS vs SAPS II (Z = 2.613, *P* = 0.009). LODS had the highest sensitivity (73.40%) and Youden’s index (0.6064), while SAPS II had the highest specificity (90.43%). The results are presented in Table 4.

**Table 3:** Comparison of ROC curves (before PSM).

Scoring system	AUC	95%CI	Optimal cut-off	Sensitivity (%)	Specificity (%)	Youden’s index
LODS	0.867	0.834 ~ 0.895	6.0	70.19	87.80	0.5799
OASIS	0.827	0.792 ~ 0.859	38.0	67.31	85.89	0.5319
SAPS II	0.894	0.864 ~ 0.919	37.0	89.42	74.40	0.6382

*AUC* area under curves, *LODS* Logistic Organ Dysfunction System, *OASIS* Oxford Acute Severity of Illness Score, *SAPS II* Simplified Acute Physiology Score.

**Table 4:** Comparison of ROC curves (after PSM).

Scoring system	AUC	95%CI	Optimal cut-off	Sensitivity (%)	Specificity (%)	Youden’s index
LODS	0.877	0.821 ~ 0.920	6.0	73.40	87.23	0.6064
OASIS	0.821	0.758 ~ 0.873	38.0	68.09	86.17	0.5426
SAPS II	0.881	0.826 ~ 0.923	47.0	69.15	90.43	0.5957

*AUC* area under curves, *LODS* Logistic Organ Dysfunction System, *OASIS* Oxford Acute Severity of Illness Score, *SAPS II* Simplified Acute Physiology Score.

We conducted a stratified analysis of the patients who underwent PCI/CABG or not. In the PCI/CABG group, the AUCs of LODS, OASIS, SAPS II were 0.853, 0.825, and 0.867, respectively (Fig. 2C). The results of AUCs comparisons were LODS vs OASIS (Z = 0.966, *P* = 0.334), LODS vs SAPS II (Z = 0.571, *P* = 0.568), and OASIS vs SAPS II (Z = 1.402, *P* = 0.161). LODS had the highest sensitivity (82.93%) and Youden’s index (0.6024), while OASIS had the highest specificity (83.39%). In the non-PCI/CABG group, the AUCs of LODS, OASIS, SAPS II were 0.857, 0.804, and 0.897, respectively (Fig. 2D). The results of AUCs comparisons were LODS vs OASIS (Z = 2.234, *P* = 0.026), LODS vs SAPS II (Z = 1.868, *P* = 0.062), and OASIS vs SAPS II (Z = 3.457, P = 0.0005). SAPS II had the highest sensitivity (95.24%) and Youden’s index (0.6190), while LODS had the highest specificity (92.38%). The results are presented in Tables 5 and 6.

**Table 5:** Comparison of ROC curves (patients underwent PCI/CABG).

Scoring system	AUC	95%CI	Optimal cut-off	Sensitivity (%)	Specificity (%)	Youden’s index
LODS	0.853	0.812 ~ 0.888	4.0	82.93	77.32	0.6024
OASIS	0.825	0.781 ~ 0.863	36.0	68.29	83.39	0.5168
SAPS II	0.867	0.828 ~ 0.901	37.0	80.49	77.00	0.5748

*AUC* area under curves, *LODS* Logistic Organ Dysfunction System, *OASIS* Oxford Acute Severity of Illness Score, *SAPS II* Simplified Acute Physiology Score.

**Table 6:** Comparison of ROC curves (patients not underwent PCI/CABG).

Scoring system	AUC	95%CI	Optimal cut-off	Sensitivity (%)	Specificity (%)	Youden’s index
LODS	0.857	0.794 ~ 0.906	8.0	65.08	92.38	0.5746
OASIS	0.804	0.736 ~ 0.861	40.0	68.25	84.76	0.5302
SAPS II	0.897	0.841 ~ 0.939	37.0	95.24	66.67	0.6190

*AUC* area under curves, *LODS* Logistic Organ Dysfunction System, *OASIS* Oxford Acute Severity of Illness Score, *SAPS II* Simplified Acute Physiology Score.

### Comparison of Decision curves

**Figure 3:**
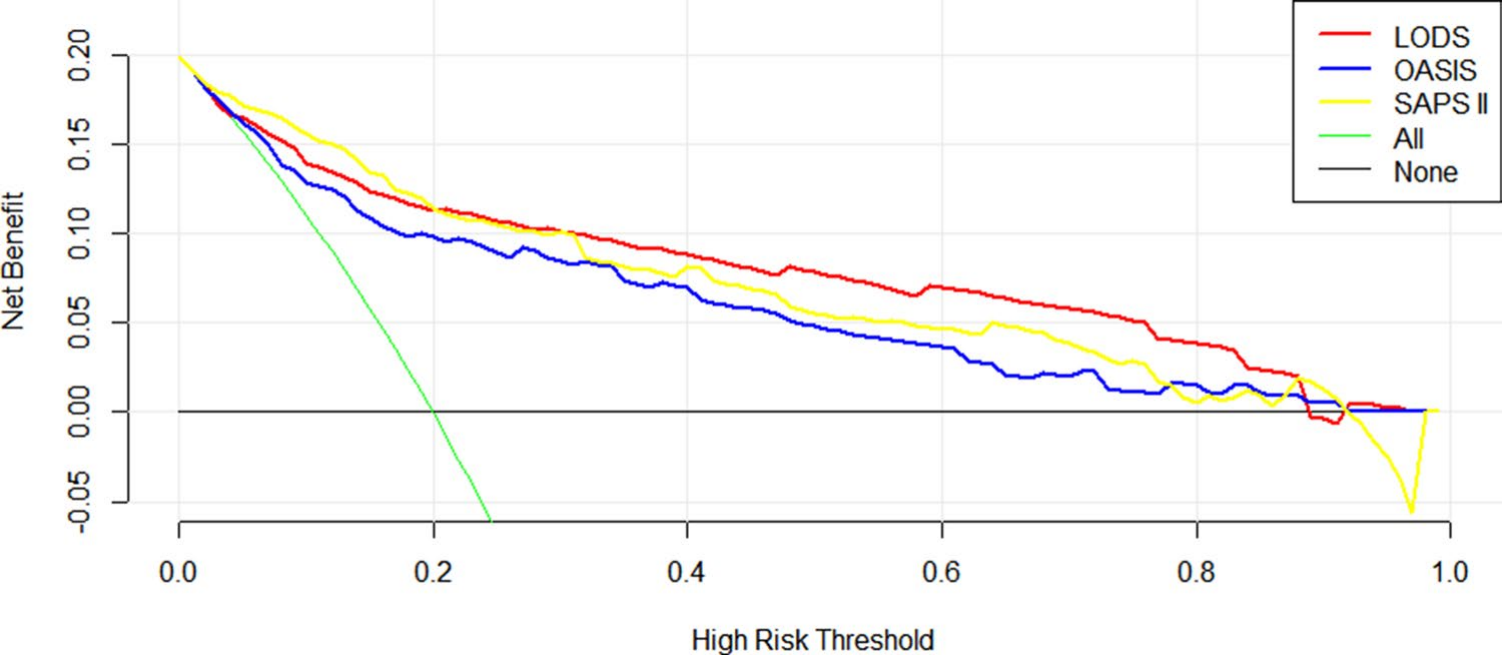
Decision curve analysis of the three scoring systems for the study cohort (before propensity score matching). X-axis indicates the threshold probability for in-hospital mortality and Y-axis indicates the net benefits.

**Figure 4:**
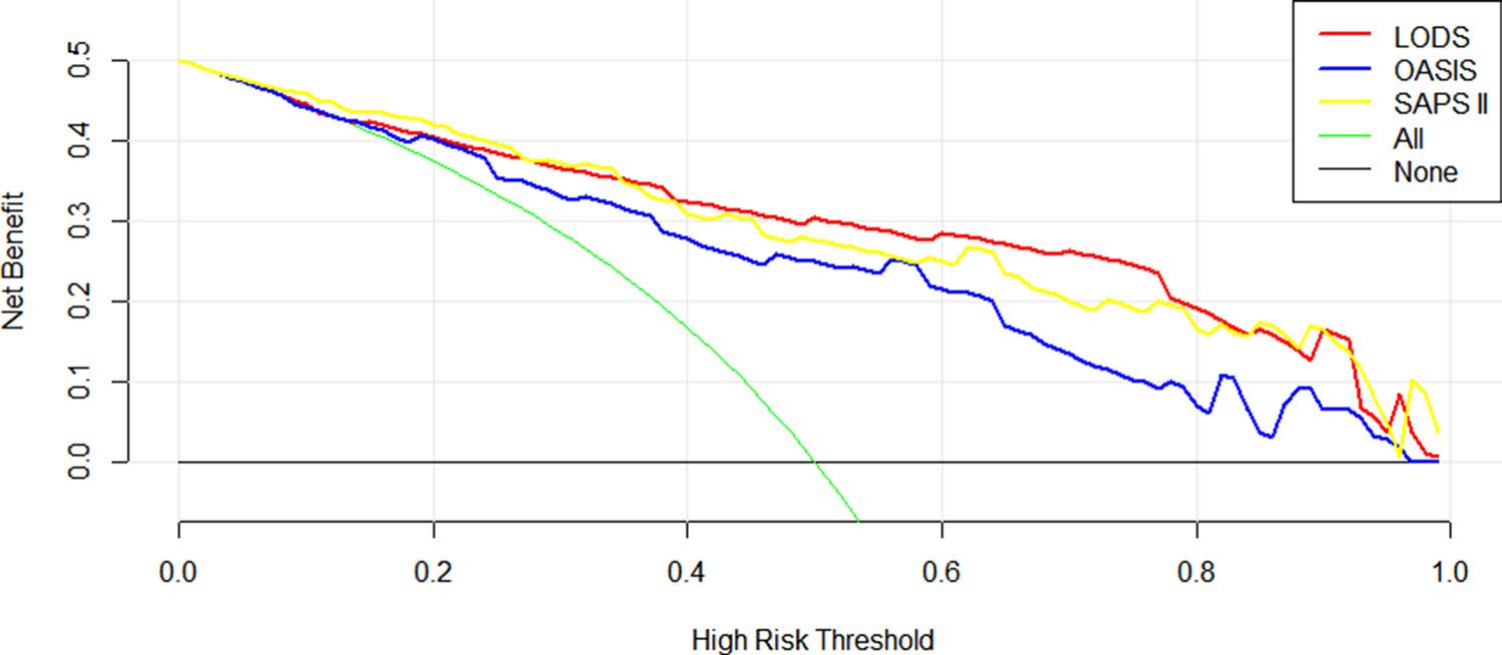
Decision curve analysis of the study cohort (after propensity score matching).

The three decision curves (the study cohort before propensity score matching, Fig. 3; after propensity score matching, Fig. 4) all showed that the red line representing LODS were almost always above the yellow line representing SAPS II and the blue line representing OASIS (in descending order were LODS, SAPS II, and OASIS), which means LODS had the greatest net benefit ranges among the three scoring systems.

## Discussion

Patients with STEMI remain at increased risk of mortality if they survive the initial ischaemic event^20^. Rapid and accurate assessment of the severity affects the treatment and prognosis of STEMI critically^21^, especially for the patients in ICU. To the best of our knowledge, our study explored the predictive value of the three scoring systems (LODS, OASIS, and SAPS II) for in-hospital mortality of intensive care STEMI patients for the first time. We confirmed that both LODS and SAPS II have good predictive value, and all AUCs were greater than 0.85. Retrospective studies may have potential biases, therefore we performed a PSM analysis and the results still suggested that LODS and SAPS II have excellent predictive value. Considering whether underwent PCI/CABG or not may have a great impact on the prognosis of the study cohort, we conducted a stratified analysis, and the results still supported the above conclusions. Comparing LODS and SAPS II, there was no significant statistical difference between the two scoring systems (*P* > 0.05 for all), and in most cases, both were higher than OASIS in predictive value.

**Figure 5:**
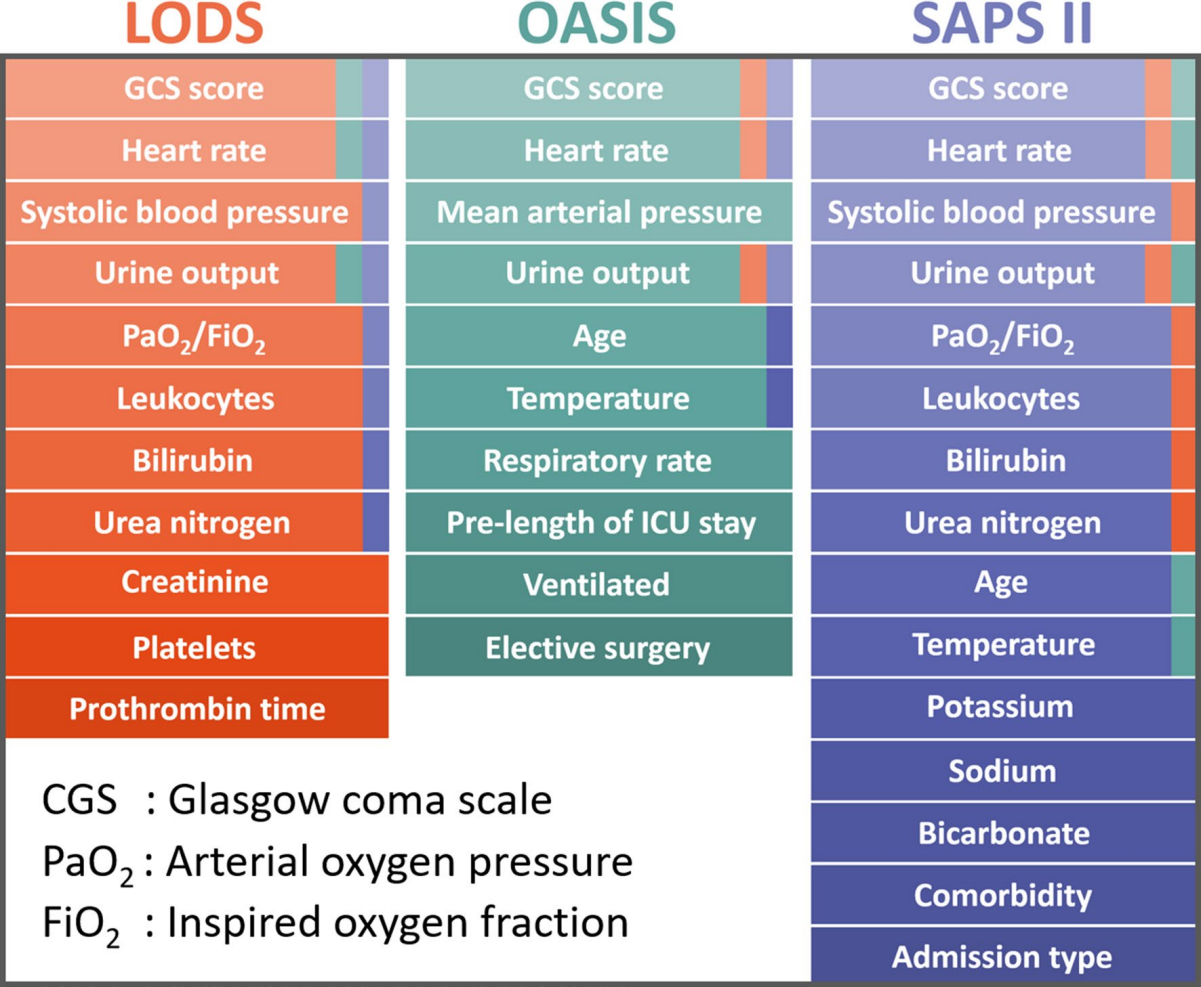
Details of the three scoring systems.

The OASIS scoring system contains 10 variables^9^, which is the least among the three scoring systems (Fig. 5). Actually, most of the intensive care patients with STEMI require emergency surgery, and the variable "elective surgery" may not apply to these patients. Meanwhile, the widespread use of PCI/CABG has significantly increased the survival rates of STEMI patients in the past decade, and most STEMI patients may not have the "pre-length of ICU stay". In addition, the focus of the treatment of STEMI patients is the recanalization of the coronary arteries and the rescue of the myocardium, while ventilation therapy is often used in the treatment of STEMI in an auxiliary form. The above factors ultimately lead to the lowest predictive value of the OASIS scoring system.

The sophisticated scoring systems require the collection of numerous physiologic measurements, making their applications in clinical practice difficult. The LODS scoring system contains 11 variables, and SAPS II contains 15 variables (Fig. 5). The common variables of LODS and SAPS II are mostly related to outcomes of STEMI. For example, the GCS score was confirmed to be higher in acute myocardial infarction patients than in acute trauma patients^22^. Furthermore, heart rate and blood pressure are undoubtedly closely related to myocardial infarction (especially in intensive care patients with STEMI), which characterizes the severity of the disease. Acute myocardial infarction is the most common cause of cardiogenic shock, and cardiogenic shock can lead to end-organ hypoperfusion (such as the kidney) and tissue hypoxia^23^. As a result, urine output and PaO_2_/FiO_2_ can reflect the severity and prognosis of myocardial infarction (including STEMI) to a certain extent correspondingly. The LODS scoring system has 4 fewer variables than SAPS II, but its predictive value is still comparable to SAPS II, which may be largely contributed by platelets and prothrombin time. Platelet activation and thrombin generation play key roles in intracoronary thrombus formation^24^. Platelets and prothrombin time largely reflect the conditions of platelet activation and thrombin generation, thus LODS is suitable for predicting the prognosis of STEMI. Although SAPS II contains many variables, electrolyte variables such as potassium, sodium, and bicarbonate tend to reflect the degree of metabolic acidosis in the form of anion gap (research showed that about 72% of critically ill patients have increased initial anion gap levels when they are admitted to the ICU, and the in-hospital mortality rate of patients with an elevated anion gap is much higher than that of patients without elevated anion gap^25^). For "comorbidity", this variable not only increases the workload when calculating scores but also may be more suitable for the prediction of long-term mortality; in addition, patients with STEMI are often admitted to the emergency department, therefore the variable "admission type" may not be sensitive and specific. In summary, LODS and SAPS II have similar predictive values, but LODS is more concise and maybe more acceptable to clinicians for application.

DCA could guide the decisions of treatment in clinical work. In our study, before/after PSM, the decision curves showed that LODS has the largest range under the curves (mainly in the high-risk threshold area between 0.4 and 0.8, Figs. 3 and 4), indicating that LODS had a slightly better net benefit in the threshold area. This is a large range of clinically reasonable preferences, therefore, considering that LODS not only has excellent predictive value for in-hospital mortality of intensive care patients with STEMI (as we discussed above) and greater clinical benefit, it may be recommended for clinical use.

Prior to this, researchers have made some explorations on the prediction of in-hospital mortality of STEMI patients. Koonsiripaiboon et al. used the Global Registry of Acute Coronary Events (GRACE) risk score to predict in-hospital mortality for 209 STEMI patients and found that the scoring system was validated^26^. Ugalde et al. predicted in-hospital mortality of ST elevation acute myocardial infarction using the Thrombolysis In Myocardial Infarction (TIMI) risk score and found that TIMI score was acceptably useful (the AUC for the ROC curve was 0.7)^27^. It should be noted that the above two scoring systems are commonly used to predict medium and long-term mortality, such as 30-day mortality, 180-day mortality, and 1-year mortality. In terms of short-term (such as in-hospital mortality) predictive value, however, there is still a lack of multi-center large sample clinical evidence. Moreover, GRACE and TIMI score are more difficult to score and require repeated evaluations, which rely heavily on dynamic electrocardiogram (ECG) changes and myocardial injury markers. The three scoring systems in this study are all applicable to ICU, and the predictive value of LODS has been confirmed. Therefore, the LODS scoring system may be more valuable for the prediction of in-hospital mortality for intensive care patients with STEMI.

There are several limitations to this study. Firstly, since it is currently impossible to extract imaging data such as ECG and coronary angiography from the MIMIC-IV database, it is difficult to obtain an accurate GRACE score and TIMI risk score. This study did not include the above two scoring systems for comparison; secondly, the database has too many missing values for some myocardial injury markers such as troponin on the first day of admission. We did not take these factors into consideration, which may affect the results; thirdly, the results of before/after PSM and stratified analysis found that the optimal cut-off values of each group were slightly different, which may be related to the variation between groups. Both patient characteristics and center characteristics may affect the results, and may even cause the scoring system to overestimate the mortality rate^28^; last but not least, the clinical strategies for patients with STEMI have changed over this relatively long time period (from 2008 to 2019), for example, from early revascularization of all vessels to revascularization of the culprit vessel only, indication for CABG etc. However, we cannot evaluate the biases caused by the changes. Since this study is a single-center study based on the American population, the conclusions of this study still need to be confirmed by well-designed prospective, multi-center clinical trials.

## Conclusions

Among the three scoring systems, LODS and SAPS II have excellent predictive value, and in most cases, both were higher than OASIS. With a more concise composition and greater clinical benefit, LODS may be a better predictor of in-hospital mortality for intensive care patients with STEMI.
